# Incidence of seroma and postoperative complications after breast surgery before and during the Covid-19 pandemic: results from a retrospective multicenter analysis

**DOI:** 10.1186/s12885-025-13425-4

**Published:** 2025-01-15

**Authors:** Maximilian Heinz Beck, Izabela A. Brachaczek, Pimrapat Gebert, Jens-Uwe Blohmer, Askin C. Kaya, Julia S. M. Zimmermann, Julia C. Radosa, Maria M. Karsten

**Affiliations:** 1 Department of Gynecology with Breast Center, Charité-Universitätsmedizin Berlin, Corporate Member of Freie Universität Berlin, Humboldt-Universität zu Berlin and Berlin Institute of Health, Charité Universitätsmedizin Berlin, Berlin, Germany; 2 Department of Gynecology with Center for Oncological Surgery, Charité-Universitätsmedizin Berlin, Corporate Member of Freie Universität Berlin, Humboldt-Universität zu Berlin and Berlin Institute of Health, Charité Universitätsmedizin Berlin, Berlin, Germany; 3 Institute of Biometry and Clinical Epidemiology, Charité – Universitätsmedizin Berlin, Corporate Member of Freie Universität Berlin and Humboldt-Universität zu Berlin, Berlin, Germany; 4 Berlin Institute of Health at Charité –Universitätsmedizin Berlin, Berlin, Germany; 5 Department of Gynecology and Obstetrics, Saarland University Hospital, Homburg, Saar, Germany; 6 Department of Gynecology with Center for Oncological Surgery, Charité-Universitätsmedizin Berlin, Berlin, Germany

**Keywords:** Seroma, Breast surgery, Covid-19, Corona Virus, Postoperative complications

## Abstract

**Background:**

In recent years, there has been a growing number of case reports documenting delayed seroma in patients with a history of breast surgery and reconstruction. The occurrence of these seromas has been associated with prior SARS-CoV-2 infection or SARS-CoV-2 vaccination. So far, there are few systematic analyses on postoperative complications in breast surgery since the emergence of the SARS-CoV-2 pandemic.

**Study design:**

We conducted a multicenter retrospective analysis to assess the incidence of postoperative complications in two major university breast care centers in Germany during the SARS-CoV-2 pandemic (August 1st, 2021, to January 31st, 2022) compared to a reference period (August 1st, 2019, to January 31st, 2020) before the pandemic.

**Results:**

A total of 987 patients were included in this retrospective analysis, with 492 patients during the SARS-CoV-2 pandemic and 495 patients in the reference period. There was no significant difference in the incidence rate of seroma after breast surgery. However, complications such as erythema, wound infection, and wound healing disorders were notably more frequent during the SARS-CoV-2 pandemic. Multivariate analysis revealed that increasing patient age, smoking, breast implant reconstruction, axillary lymph node dissection, and previous radiation were significant clinical risk factors for seroma development.

**Conclusion:**

While our findings did not indicate an elevated incidence of seroma during the SARS-CoV-2 pandemic, we observed increased rates of erythema, wound healing disorders, and wound infection. Additional real-world evidence is needed for understanding both early and late complications following breast surgery in the context of the ongoing SARS-CoV-2 endemic.

**Supplementary Information:**

The online version contains supplementary material available at 10.1186/s12885-025-13425-4.

## Introduction

Development of breast and axillary seroma is one of the most frequently observed complications after breast surgery. Although many seromas are considered to be clinically insignificant, the presence of seroma after breast surgery is linked to an increased risk of wound infection, postoperative hematoma, long-term breast induration and unfavorable cosmesis [1]. Clinically significant breast seromas may necessitate repeated needle aspirations, which can cause patient discomfort, carry a risk of infection, and demand considerable expertise and skill from the treating surgeon [2]. Seroma after breast cancer surgery is more often observed after mastectomy compared to breast conserving surgery and incidences vary due to procedure type and assessment method between 5.4% and 43.6% [1, 3–7]. Beside the amount of resected breast tissue, the extent of axillary surgery represents a major risk factor for seroma development [5, 7]. Further reported factors influencing the development of seroma are patient age, obesity, diabetes mellitus, morbidity defined by the ASA-Score (ASA - American Society of Anesthesiologists), breast size or the presence and number of malignant nodes of the axilla [3, 7–10]. Seroma occurring immediately after surgery can be distinguished from so-called late seroma, that can be observed in 1–2% of patients after breast implant reconstruction or augmentation and occur at a longer time interval after surgery [11, 12]. Late seroma is defined as a seroma developing more than 12 months after breast surgery. Its occurrence requires thorough evaluation due to potential associations with Breast Implant-Associated Anaplastic Large Cell Lymphoma [13]. Despite the high incidence and clinical relevance, the etiology of seroma remains poorly understood.

Lately, an increasing number of case reports of pronounced late seroma in patients with breast implants after SARS-CoV-2 infection or SARS-CoV-2 vaccination emerged [8, 14–16]. Some authors hypothesized that the cumulated occurrence of late seromas could be related to SARS-CoV-2 infection or be caused as a vaccine side effect and implied thereby an immune-mediated pathogeneses of seroma [17]. So far, no systematic analysis of seroma incidence during the SARS-CoV-2 pandemic has been reported. We therefor conducted this observational retrospective study to determine the incidence of seroma and complications after breast surgery and to evaluate possible correlations to recent SARS-CoV-2 infection or SARS-CoV-2 vaccination.

## Methods

### Objectives and end points

We conducted an observational retrospective study in the breast units of two major German university hospitals. The primary endpoint was to determine possible correlations between SARS-CoV-2 infection or SARS-CoV-2 vaccination by comparing the incidence of seroma development after breast surgery before and during the pandemic. Furthermore, temporal correlations between SARS-CoV-2 infection or SARS-CoV-2 vaccination and breast seroma were assessed. Secondary endpoints included assessing the association of clinically relevant risk factors with seroma incidence and the timing of seroma development. These factors included age, body mass index (BMI), comorbidities (such as diabetes mellitus, autoimmune diseases, allergies, and smoking), prior therapies (neoadjuvant, antibody-based, and radiation), and the type of surgery performed.

The study was approved by the local ethics committee of the Charité university hospital of Berlin (EA2/155/22).

### Patients and data collection

Patients who underwent breast surgery in the certified breast centers of the Charité university hospital of Berlin and of the Saarland university hospital were included in this study. The first investigation period reached from August 1st 2021 to January 31th 2022. During this period the German vaccination campaign against SARS-CoV-2 recommended the second booster vaccination to the general population and daily administered vaccinations against SARS-CoV-2 reached new daily heights. Furthermore, the spread of the delta and early omicron variant were observed in Germany during that episode. The seconded observation period reached from August 1st 2019 to January 31th 2020 and served as a reference period before the SARS-CoV-2 outbreak in Germany. All patients after breast surgery in the participating institutions were included during the above-mentioned periods. There were no restrictions regarding indication or extent of surgery. Surgical Procedures included breast-conserving techniques as well as skin- or nipple-sparing mastectomy and total mastectomy. Patient characteristics as well as disease and treatment information were retrospectively collected from the electronic medical records (EMR) at each hospital.

In case of documented postoperative seroma, we assessed the date of first documentation, number of repeated aspirations and amount of drained seroma. Postoperative seroma was defined as requiring a puncture post drain removal. Data about SARS-CoV-2 infection and vaccination was only assessed for the investigation period during the SARS-CoV-2 pandemic. The collected information about SARS-CoV-2 vaccination status included date and number of vaccinations, type of vaccine and vaccine manufacturer and were pulled from the EMR where available. In case of missing information, we contacted the patient directly via phone to obtain the dates and types of vaccinations and/or infections they had. In case of SARS-CoV-2 infection the date of the infection defined by positive PCR was documented. In addition, we collected for both observations periods information regarding the type of surgery, extent of axillary surgery, neoadjuvant chemotherapy before surgery, sex, age, body-mass-index (BMI), history of smoking, history of diabetes mellitus, previous antibody-treatment, previous radiation of the operated breast, history of autoimmune disease, history of immunosuppression and presence of allergy as detailed in Table 1.

### Data analysis

Statistical analysis was performed using Stata IC15 (StataCorp, 2017, College Station, TX, USA). The incidence of seroma was reported as a percentage, and a 95% confidence interval (CI) was estimated. We performed the Chi-square test for comparing the incidence of seroma and the Mann-Whitney U test for comparing the volume of drained seroma before and during the SARS-CoV-2 pandemic. Multivariable analysis was performed when considering the seroma as a binary outcome using multiple logistic regression. As well, we considered the time-to-event analysis by defining the time between the date of operation and the date of seroma. The censors are defined as patients who did not develop postoperative seroma until March 31st, 2020, and March 31st, 2022, respectively, before and during the SARS-CoV-2 pandemic. The analysis is performed to adjust for predefined covariate factors, e.g., age, sex and related comorbidities, previous therapy, type of surgery. A p value < 0.05 was considered to indicate statistical significance without adjustment for multiple testing. All p values constitute exploratory data analysis. Descriptive statistics are presented as the mean, standard deviation, median, interquartile range or absolute and relative frequencies depending on scale.

## Results

### Patient characteristics

Overall, 987 patients were enrolled in this retrospective analysis, of which 492 patients were included in the investigation period during the SARS-CoV-2 pandemic (August 1st 2021 to January 31th 2022) and 495 patients in the reference period before the SARS-CoV-2 pandemic (August 1st 2019 to January 31th 2020). More patients were included at the breast center of Charité due to higher patient volume.

All patients underwent surgical treatment. Breast conserving surgery was the most common performed procedure before (*n* = 304; 61.41%) and during the SARS-CoV-2 pandemic (*n* = 286; 58.13%). Axillary surgery was undertaken in about half of the patients and implant-based breast reconstructions were performed to a similar extent during and before the pandemic. The most frequent indication of surgery was invasive carcinoma of no special type. Prior to the SARS-CoV-2 pandemic, more patients underwent surgery for carcinoma in situ. During the pandemic, a higher proportion of male patients were treated. Other patient characteristics were similarly distributed between the two cohorts. A more detailed overview of patient characteristics is displayed in Table 1.

**Table 1:** Patient’s characteristics before and during the SARS-CoV-2 pandemic. N = number

Patient´s characteristics		Before SARS- CoV-2 pandemic (*n* = 495)	During SARS- CoV-2 pandemic (*n* = 492)	*p*-value
Study center	Charite	290 (58.59%)	307 (62.40%)	0.221
	Homburg	205 (41.41%)	185 (37.60%)	
Age (years, mean (SD))		52.68 (19.99)	54.77 (14.80)	0.062
Sex	Male	5 (1.01%)	15 (3.05%)	**0.023**
	Female	490 (98.99%)	477 (96.95%)	
BMI (kg/m^2^), mean (SD)		25.94 (5.28)	25.94 (5.52)	0.995
Type of Surgery	Breast- conserving Surgery	304 (61.41%)	286 (58.13%)	0.293
	Subcutaneous Mastectomy	48 (9.70%)	55 (11.18%)	0.446
	Mastectomy	55 (11.11%)	55 (11.18%)	0.973
	Breast Implant Reconstruction	81 (16.36%)	83 (16.87%)	0.820
	Sentinel lymph node biopsy	210 (42.42%)	202 (41.06%)	0.663
	Axillary lymph node dissection	52 (10.51%)	60 (12.20%)	0.403
	other	71 (14.34%)	67 (13.62%)	0.742
Indication of Surgery	Invasive carcinoma of no special type	266 (53.74%)	273 (55.49%)	**0.003**
	Ductal carcinoma in situ	63 (12.73%)	28 (5.69%)	
	Invasive Lobular Carcinoma	42 (8.48%)	33 (6.71%)	
	Lobular carcinoma in situ	3 (0.61%)	-	
	other	156 (31.52%)	158 (32.11%)	
Previous Therapy	Neoadjuvant Therapy	70 (14.14%)	82 (16.67%)	0.272
	Radiation	53 (10.71%)	37 (7.52%)	0.082
	Antibody-Based	22 (4.44%)	30 (6.10%)	0.362
Comorbidities	Diabetes mellitus	31 (7.03%)	46 (9.35%)	0.199
	Smokers	48 (10.88%)	61 (12.40%)	0.472
	Autoimmune diseases	26 (5.92%)	17 (3.46%)	0.073
	Allergies	107 (43.85%)	32 (35.56%)	0.172
Previous Breast Operation		156 (32.23%)	155 (31.63%	0.841
Previous SARS- CoV-2 Infection			65 (13.2%)	
Previous SARS- CoV-2 Vaccination			392 (79.7%)	
Patient´s characteristics		Before SARS- CoV-2 pandemic (*n* = 495)	During SARS- CoV-2 pandemic (*n* = 492)	p-value
Study center	Charite	290 (58.59%)	307 (62.40%)	0.221
	Homburg	205 (41.41%)	185 (37.60%)	
Age (years, mean (SD))		52.68 (19.99)	54.77 (14.80)	0.062
Sex	Male	5 (1.01%)	15 (3.05%)	**0.023**
	Female	490 (98.99%)	477 (96.95%)	
BMI (kg/m^2^), mean (SD)		25.94 (5.28)	25.94 (5.52)	0.995
Type of Surgery	Breast- conserving Surgery	304 (61.41%)	286 (58.13%)	0.293
	Subcutaneous Mastectomy	48 (9.70%)	55 (11.18%)	0.446
	Mastectomy	55 (11.11%)	55 (11.18%)	0.973
	Breast Implant Reconstruction	81 (16.36%)	83 (16.87%)	0.820
	Sentinel lymph node biopsy	210 (42.42%)	202 (41.06%)	0.663
	Axillary lymph node dissection	52 (10.51%)	60 (12.20%)	0.403
	other	71 (14.34%)	67 (13.62%)	0.742
Indication of Surgery	Invasive carcinoma of no special type	266 (53.74%)	273 (55.49%)	**0.003**
	Ductal carcinoma in situ	63 (12.73%)	28 (5.69%)	
	Invasive Lobular Carcinoma	42 (8.48%)	33 (6.71%)	
	Lobular carcinoma in situ	3 (0.61%)	-	
	other	156 (31.52%)	158 (32.11%)	
Previous Therapy	Neoadjuvant Therapy	70 (14.14%)	82 (16.67%)	0.272
	Radiation	53 (10.71%)	37 (7.52%)	0.082
	Antibody-Based	22 (4.44%)	30 (6.10%)	0.362
Comorbidities	Diabetes mellitus	31 (7.03%)	46 (9.35%)	0.199
	Smokers	48 (10.88%)	61 (12.40%)	0.472
	Autoimmune diseases	26 (5.92%)	17 (3.46%)	0.073
	Allergies	107 (43.85%)	32 (35.56%)	0.172
Previous Breast Operation		156 (32.23%)	155 (31.63%	0.841
Previous SARS- CoV-2 Infection			65 (13.2%)	
Previous SARS- CoV-2 Vaccination			392 (79.7%)	

### Incidence of seroma

**Fig. 1:**
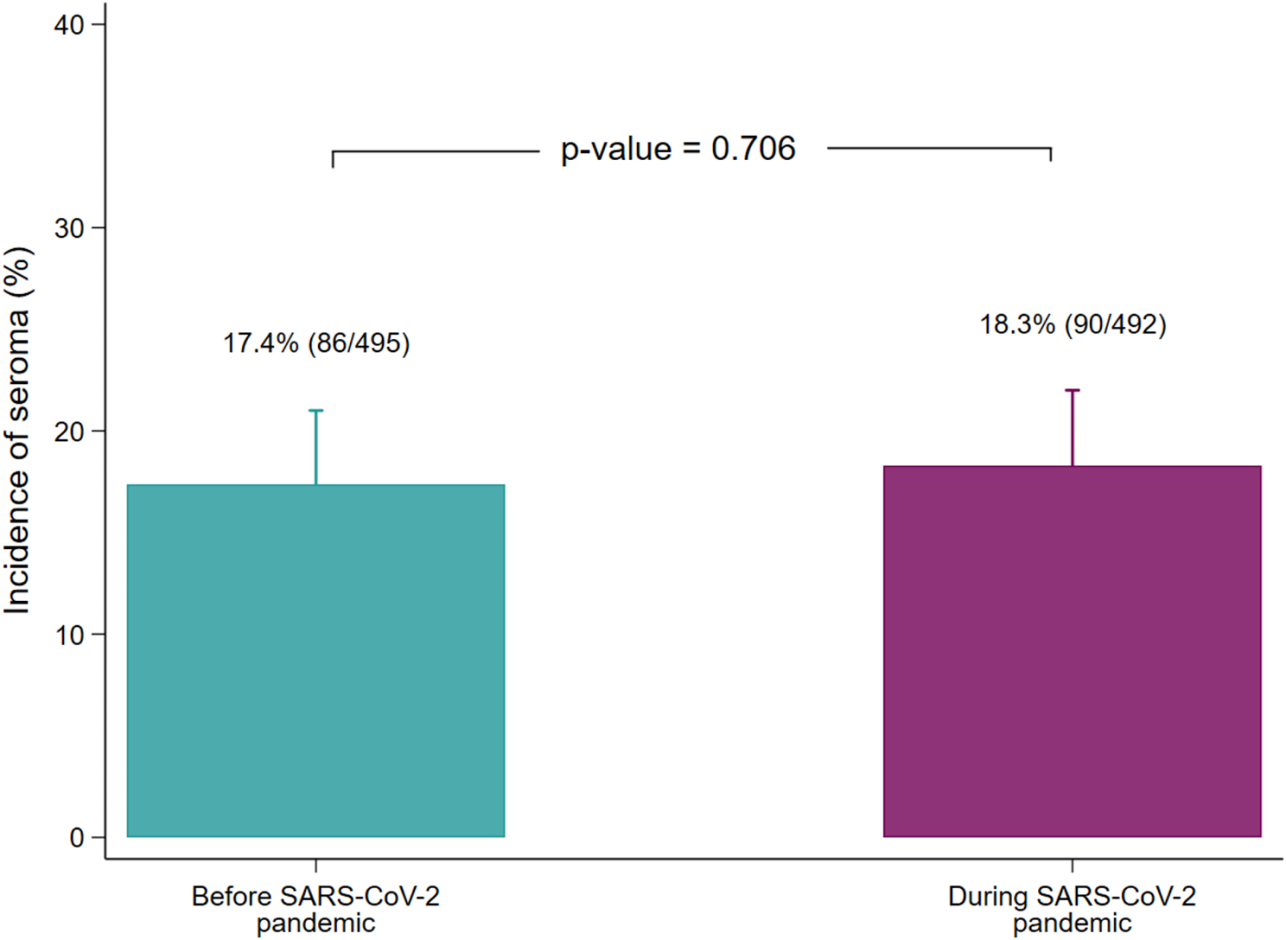
Incidence of seroma (%). Error bar indicates 95% confidence interval. The number of patients is displayed in parentheses behind the percentage

The overall occurrence rate of seroma was 17.83% during the combined observation period (*n* = 176). We could observe no relevant difference between the incidence of seroma during the SARS-CoV-2 pandemic (18.29%, 90 of 492; 95%CI: 14.97 − 22.00%) and during the reference period before the SARS-CoV-2 pandemic (17.37%, 86 of 495; 95%CI: 14.14 − 21.00%; *p* = 0.706). Figure 1 presents the incidence of seroma.

In a further analysis, incidence of seroma was compared between patients with and without SARS-CoV-2 vaccination during the SARS-CoV-2 pandemic. Higher rates of seroma were observed in patients without SARS-CoV-2 vaccination (35.3%; 6 of 17) in comparison with patients with SARS-CoV-2 vaccination (18.9%; 74 of 392). However, the differences did not reach statistical significance (*p* = 0.095) due to the limited number of patients without a vaccination. The SARS-CoV-2 vaccination status of 83 patients (16,9%; 83 of 492) was unknown.

Due to the limited number of patients with previous SARS-CoV-2 infection, statistical testing was not conducted. It is noteworthy that none of the patients who had previously experienced SARS-CoV-2 infection (*n* = 14) developed postoperative seroma.

### Seroma volume

In cases of clinically significant seroma, an average of 2.1 ± 1.6 (range: 1 to 9) repeated needle aspirations were performed. The number of repeated aspirations did not differ significantly between the periods before and during the pandemic (*p* = 0.44). Additionally, no differences were observed in the management of seromas based on the type of surgery or implant reconstruction. Two patients required a secondary surgery for seroma evacuation. The volume of drained seroma was higher during the SARS-CoV-2 pandemic (median volume: 98 ml; IQR 23–225) in comparison to the pre-pandemic period (median volume 52 ml; IQR 20–160). However, the difference was not found to be statistically significant (*p* = 0.17).

### Incidence of other complications

Table 2 presents a comparison of the incidences of various postoperative complications in patients before and during the SARS-CoV-2 pandemic. The complications analyzed include seroma, erythema, wound infection, wound dehiscence, hematoma, and wound healing disorders. The incidence of erythema significantly increased during the pandemic, rising from 20 out of 495 patients (4.09%) pre-pandemic to 39 out of 492 patients (7.93%) during the pandemic (*p* = 0.011). Similarly, wound infection rates increased from 1.63% (*n* = 8) pre-pandemic to 4.67% (*n* = 23) during the pandemic (*p* = 0.006), whereas wound dehiscence rates decreased from 2.63% (*n* = 13) to 0.81% (*n* = 4; *p* = 0.029). Wound healing disorders also became more prevalent, increasing from 0.61% (*n* = 3) to 3.25% (*n* = 16; *p* = 0.002). A total of 19 patients required secondary surgical intervention due to postoperative complications. The reasons for these interventions included hematoma evacuation (*n* = 8), abscess drainage (*n* = 4), wound dehiscence repair (*n* = 4), seroma evacuation (*n* = 2), and debridement (*n* = 1).

In contrast, the incidence of seroma and hematoma showed no significant difference between the two periods, with p-values of 0.71 and 0.16, respectively.

**Table 2:** Incidence of complications during the observation periods

Complications	Before SARS-CoV-2 pandemic (*n* = 495)	During SARS-CoV-2 pandemic (*n* = 492)	*p*-value
Seroma	86 (17.37%)	90 (18.29%)	0.71
Erythema	20 (4.09%)	39 (7.93%)	0.011
Wound infection	8 (1.63%)	23 (4.67%)	0.006
Wound dehiscence	13 (2.63%)	4 (0.81%)	0.029
Hematoma	20 (4.04%)	12 (2.44%)	0.16
Wound healing disorder	3 (0.61%)	16 (3.25%)	0.002

*Multivariable analysis for seroma incidence.*

The bivariate analysis identified increasing age, smoking, breast implant reconstruction, axillary lymph node dissection, prior neoadjuvant therapy, prior antibody treatment, and previous radiation therapy as factors significantly associated with an elevated risk of breast seroma. Conversely, previous breast surgery, known autoimmune or allergic conditions, diabetes mellitus, body mass index (BMI), and the SARS-CoV-2 pandemic did not demonstrate a statistically significant association with the development of breast seroma. Detailed results are provided in Supplementary Table A1.

For the multivariate analysis, only clinical variables achieving statistical significance in the bivariate analysis (*p* < 0.05) were considered, along with the inclusion of the SARS-CoV-2 pandemic as a variable of interest. The results of the multivariate logistic regression, evaluating the association between seroma incidence, the SARS-CoV-2 pandemic, and other clinical parameters, are summarized in Table 3.

Table 3 shows the results of the multivariable analysis using a logistic regression model for exploring an association between seroma incidence and the SARS- CoV- 2 Pandemic and other clinical factors. We only considered clinical variables, that reached significance in the bivariate analysis (with a p-value < 0.05) for the multivariate testing. Results from the bivariate analysis can be found in Supplementary Table A1.

No significant correlation between the SARS-CoV-2 pandemic and the occurrence of postoperative seroma was found in the multivariable analysis, although we could observe a slightly increase of the risk for seroma development during the pandemic (OR_adjusted_ 1.18; 95%CI: 0.82–1.69). As well documented in previous studies, increasing patient age, smoking, breast implant reconstruction, axillary lymph node dissection and previous radiation were significant clinical risk factors for the development of seroma [3, 7–10].

**Table 3:** Multivariable analysis using multiple logistic regression for seroma incidence. OR = odds ratio

Factors	Multivariable analysis (*n* = 923)	
	Adjusted OR (95%CI)	*p*-value
SARS- CoV- 2 Pandemic	1.18 (0.82–1.69)	0.382
Age (years):		
< 40	1 (Reference)	
40–49	1.84 (0.90–3.77)	0.096
50–59	2.32 (1.16–4.63)	0.017
60+	2.88 (1.47–5.65)	0.002
- Smokers	1.95 (1.19–3.20)	0.008
Breast Implant Reconstruction	2.05 (1.27–3.29)	0.003
Axillary lymph node dissection	3.30 (2.02–5.39)	< 0.001
Sentinel lymph node biopsy	3.80 (2.37–6.07)	< 0.001
Previous Therapy: - Neoadjuvant Therapy	1.21 (0.71–2.06)	0.475
- Antibody-Based	1.37 (0.65–2.89)	0.409
- Radiation	2.00 (1.15–3.47)	0.014

### Time to seroma analysis

**Fig. 2:**
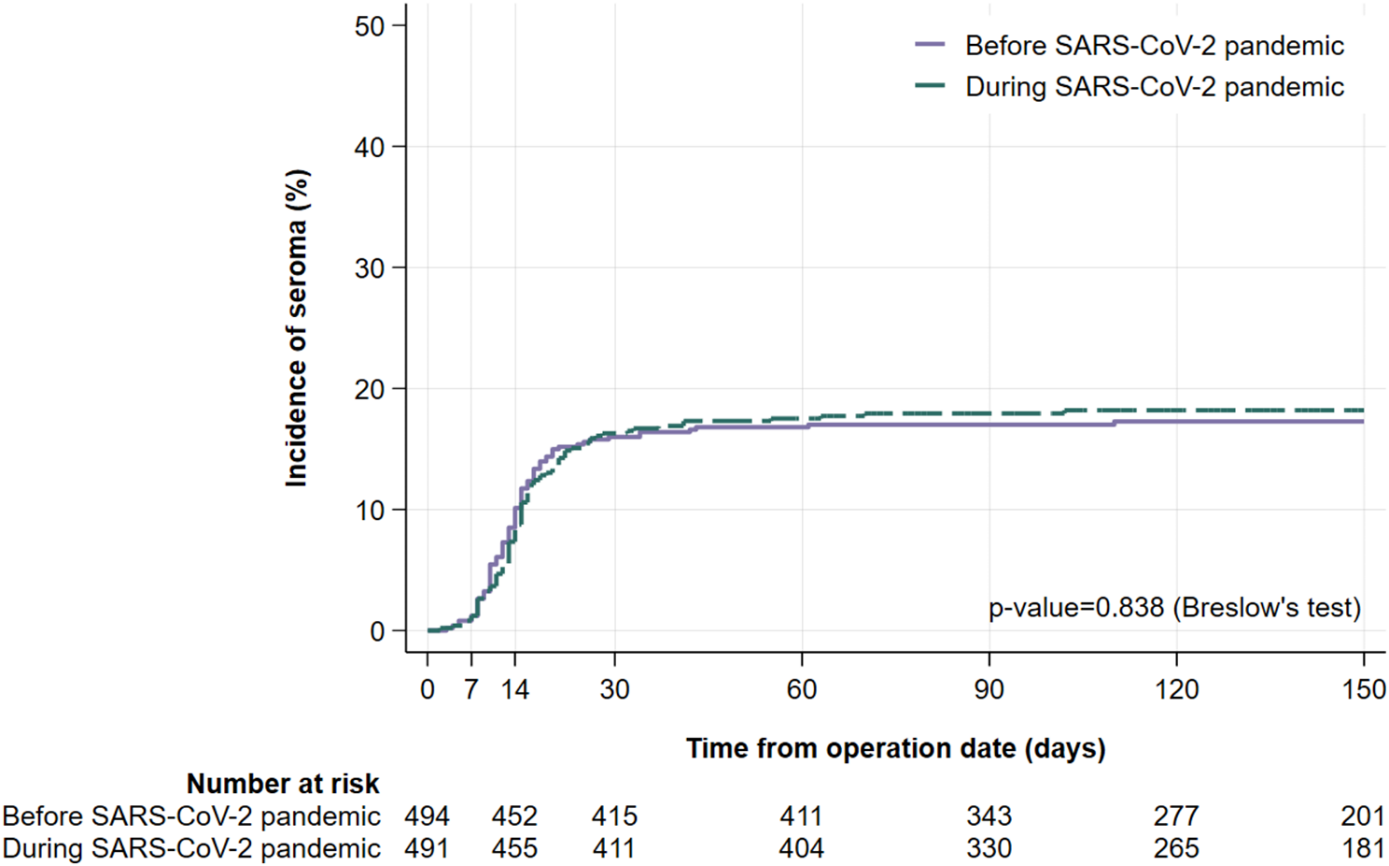
Time to event (seroma development) analysis. Blue line represents seroma incidence before the SARS-CoV-2 pandemic, red line during the SARS-CoV-2. Two cases were unknown the date of seroma development

We conducted a time-to-event (seroma development) analysis to explore whether there were differences in the dynamic of seroma occurrence before and during the SARS-CoV-2 pandemic. Figure 2 illustrates the occurrence of seroma. No significant difference of the incidence rate of seroma formation was observed 14 days and respectively 30 days after from surgery comparing the two observation periods. Before the SARS- CoV-2 pandemic the seroma incidence 14 days after surgery was 10.59% (95% CI: 8.03–13.98) and 17.32% (95% CI: 13.89–21.59) after 30 days. During the SARS- CoV-2 pandemic, the incidence of seroma was 9.11% (95% CI: 6.76–12.28) after 14 days and 17.68% (95% CI: 14.20–22.02) 30 days after surgery.

### Multivariable analysis for time the time to seroma development

A multivariable analysis using a Cox regression model was used to assess the impact of covariates on the time until seroma formation (Time-to-event analysis, see Table 4). Again, we only considered clinical variables, that reached significance in the bivariate analysis (with a p-value < 0.05) for the multivariate testing. Results from the bivariate analysis can be found in supplementary Table A2.

No association between the time after breast surgery until development of seroma and the SARS-CoV-2 pandemic was observed (HR_adjusted_= 1.14; 95% CI: 0.82–1.57). Increasing patient age, axillary lymph node dissection, implant reconstruction and previous radiation were associated with earlier onset of seroma development.

**Table 4:** Multivariable analysis using Cox regression model for Time-to-Seroma formation. HR = hazard ratio

Factors	Multivariable analysis (*n* = 921)	
	Adjusted HR (95%CI)	*p*-value
SARS- CoV- 2 Pandemic	1.14 (0.82–1.57)	0.440
Age (years):		
< 40	1 (Reference)	
40–49	1.70 (0.88–3.28)	0.116
50–59	2.38 (1.27–4.47)	0.007
60+	3.07 (1.66–5.69)	< 0.001
- Smokers	1.43 (0.93–2.18)	0.100
Breast Implant Reconstruction	1.53 (1.02–2.28)	0.039
Axillary lymph node dissection	2.96 (1.98–4.42)	< 0.001
Previous Therapy: - Neoadjuvant Therapy	1.21 (0.78–1.89)	0.391
- Antibody-Based	1.19 (0.65–2.17)	0.572
- Radiation	1.61 (1.02–2.54)	0.041

## Discussion

### Summary of results

This retrospective, cross-sectional study sought to investigate changes of seroma incidence and postoperative complications after breast surgery during the SARS-CoV2 pandemic. For this purpose, we compared the postoperative course and incidence of complications, mainly seromas in two periods before and during the SARS-CoV2 pandemic. We found no difference in the incidence of postoperative seroma before and during the SARS-CoV2 pandemic, also no relation was found between postoperative seroma and SARS-CoV2 vaccination or infection. In contrast, significantly higher rates of erythema, wound healing disorders and wound infection were found during the SARS-CoV2 pandemic. This indicates a notable shift in postoperative complications associated with the pandemic period. Meanwhile higher rates of wound dehiscence were reported before the pandemic. In the performed multivariate analysis for seroma development, we identified increasing age, breast reconstruction, radiation and axillary dissection as risk factors for the development of seroma. In contrast, previous breast surgery, neoadjuvant chemotherapy, prior antibody therapy, known autoimmune or allergic conditions, diabetes mellitus, body mass index (BMI), and smoking were not associated with an increased risk of seroma development.

### Comparison of the results with published evidence

The observed average incidence of postoperative seroma development at 17.8% aligns with previously reported rates, ranging from 6 to 36.6% for breast-conserving surgery, 14.2–43.6% for mastectomy, and 5.4% for aesthetic prosthetic surgery [1, 3, 4, 6]. Consistent with existing data, our study identified elevated seroma rates after immediate breast reconstruction and axillary dissection [7]. In line with a recent meta-analysis involving 134,191 patients, which found no correlation between prior neoadjuvant chemotherapy and seroma incidence, our findings did also not demonstrate changes in seroma incidence after previous neoadjuvant therapy [18]. Our study identified no association between prior breast surgery and the occurrence of breast seroma. To our knowledge, evidence on this specific question remains scarce. Notably, a Japanese review examining previous breast interventions found no significant relationship between prior biopsies and the development of seroma [19].

During the SARS-CoV2 pandemic, our analysis showed significant increases in postoperative complications in breast surgery, such as erythema, wound infection, and impaired wound healing. This contrasts with findings from two retrospective analyses that reported no rise in postoperative complications during the pandemic [20, 21]. However, our results align with a Dutch dual-center retrospective analysis, which reported increased susceptibility to impaired wound healing following reconstructive breast surgery during the pandemic [22]. This discrepancy highlights the variability in outcomes across different healthcare systems and patient populations. It is plausible that pandemic-related disruptions in routine healthcare, including reduced perioperative monitoring and delayed follow-up care, might have played a major role in these heightened complication rates. In addition to a reduction in surgical capacities, the length of hospital stays for patients was significantly shortened during the pandemic at our institutions. This factor may partially account for the increased rate of wound infections. The authors are not aware of any other changes or adaptations of treatment standards during the pandemic. Additionally, SARS-CoV-2 infection related factors such as pronounced inflammatory response and disrupted coagulation pathways [23] might also play a role in impaired wound healing and increased susceptibility to infections.

Nevertheless, the underlying causes for these increased postoperative complications remain speculative and underline the need for further research. Larger, multicenter studies are needed to clarify the extent of these shifts in complication rates and to determine whether they persist into the endemic phase of SARS-CoV-2.

### Seroma and SARS-CoV-2 infection

In contrast to recently published case reports of possible SARS-CoV-2 infection or vaccination related periprosthetic breast seroma, our data did not show an increase of seroma formation during the SARS-CoV-2 pandemic or any correlation between SARS-CoV-2 vaccination or infection and seroma development after breast surgery. So far 8 cases of periprosthetic breast complications with a temporal association to a recent SARS-CoV-2 vaccination were reported [14, 16, 17, 24]. These cases included erythema and swelling, seroma and rapid development of capsular contraction, each with a short temporal association to a recent SARS-CoV-2 vaccination [14, 16, 17]. Similar unpublished cases were observed in our own department leading to this retrospective analysis. There has been also one case reports of periprosthetic late seroma after SARS-CoV-2 vaccination in a patient with history of TRAM-flap and abdominal mesh reconstruction [25]. Notably, a rapid SARS-CoV-2 antigen swap of the drained fluid tested positive in this individual. Cases of periprosthetic late seroma and one case of periprosthetic multicentric breast abscess formation [26] with a short temporal correlation to a recent active SARS CoV-2 disease were reported as well in the literature. Due to the short temporal correlation, a vaccine or infection triggered immune-response with the breast prosthesis as subject was discussed [15]. This hypothesis was questioned by the fact that breast swelling and asymmetry after SARS-CoV-2 vaccination was also reported in patients without previous breast surgery [27].

Our results seem to contradict these cases as we could not demonstrate an association between SARS-CoV-2 infection or vaccination and the incidence of seroma. This might be partly explained by the fact that we mainly assessed the occurrence of early postoperative seroma, while most reported periprosthetic seroma were late seroma. Late seroma are defined as periprosthetic fluid collection which occur at least one year after implantation with a reported incidence of 0.88% [28]. Due to the low incidence of late seroma, our analysis is likely underpowered to detect differences in the occurrence of this relatively rare complication. Late seroma mostly occur with the use of textured implants [28]. Although the underlying pathological mechanisms are not well understood, they likely differ from the pathogenesis of early seroma. Nevertheless, if SARS-CoV-2 vaccine or infection triggered immune responses against (breast) implants are causative for seroma formation, we would also expect higher rates of early seroma, especially in patients with breast implants.

Furthermore, we want to outline, that the number of reported cases of seroma with possible SARS-CoV-2 association is very limited so far. Even though many cases have probably not been published, it is stochastically difficult to show possible correlations between (periprosthetic) breast seroma and SARS-CoV-2 considering the billions of vaccinated or infected individuals during the pandemic. Nevertheless, systematic analyses with appropriate cohorts are needed to evaluate the incidence of late complications and possible correlations to SARS-CoV-2 vaccination or infection. So far, the association between SARS-CoV-2 vaccination or infection and seroma development remains speculative in view of million successfully administered vaccines with acceptable safety profiles [29].

## Conclusion

A significant proportion of patients (18%) developed postoperative seromas requiring needle aspiration, underscoring the need for improvements in clinical standards.

Throughout the observation period during the SARS-CoV-2 pandemic, we found no evidence of elevated rates of seroma development. However, we did identify increased occurrences of erythema, wound healing disorders, and wound infection during the SARS-CoV-2 pandemic. Additional real-world evidence is needed for understanding both early and late complications following breast surgery, particularly in the context of the ongoing SARS-CoV-2 endemic and vaccination programs.

## Electronic supplementary material

Below is the link to the electronic supplementary material.

Supplementary Material 1

Supplementary Material 2

## Data Availability

The data presented in this study are available on request from the corresponding author.
