# Supplementary material for: Incidence of seroma and postoperative complications after breast surgery before and during the Covid-19 pandemic: results from a retrospective multicenter analysis
Source: BMC Cancer. 2025 Jan 15;25:91. doi: 10.1186/s12885-025-13425-4 (PMC11737024; doi:10.1186/s12885-025-13425-4)
Supplement: Supplementary file 1 — Supplementary Material 1 [file 12885_2025_13425_MOESM1_ESM.docx]

**Supplementary Table A1.:** Bi- and Multivariable analysis using multiple logistic regression for seroma incidence

| Factors | Bivariate analysis | | Multivariable analysis (n = 923) | |
| --- | --- | --- | --- | --- |
|  | **Crude OR (95%CI)** | **p-value** | **Adjusted OR (95%CI)** | **p-value** |
| SARS- CoV- 2 Pandemic | 1.06 (0.77- 1.48) | 0.706 | 1.18 (0.82 - 1.69) | 0.382 |
| Age (years): |  |  |  |  |
| <40 | 1 (Ref.) |  | 1 (Ref.) |  |
| 40-49 | 2.05 (1.06 - 3.98) | 0.033 | 1.84 (0.90 - 3.77) | 0.096 |
| 50-59 | 2.52 (1.34 - 4.75) | 0.004 | 2.32 (1.16 - 4.63) | 0.017 |
| 60+ | 2.41 (1.32 - 4.40) | 0.004 | 2.88 (1.47 - 5.65) | <0.002 |
| Body- Mass- Index (BMI): |  |  |  |  |
| <25 | 1 (Ref.) |  |  |  |
| 25<=BMI<30 | 1.34 (0.92- 1.95) | 0.131 |  |  |
| >=30 | 1.39 (0.91- 2.13) | 0.128 |  |  |
| Previous Breast Surgery | 1.21 (0.86- 1.72) | 0.274 |  |  |
| Comorbidities:  - Diabetes mellitus | 1.63 (0.94- 2.83) | 0.079 |  |  |
| - Smokers | 2.10 (1.33- 3.32) | 0.001 | 1.95 (1.19 - 3.20) | 0.008 |
| - Autoimmune disease | 1.49 (0.72- 3.09) | 0.283 |  |  |
| - Allergies | 1.08 (0.64- 1.83) | 0.780 |  |  |
| Breast Implant Reconstruction | 1.68 (1.13- 2.51) | 0.010 | 2.05 (1.27 - 3.29) | 0.003 |
| Axillary lymph node dissection | 3.74 (2.45- 5.71) | <0.001 | 3.30 (2.02 - 5.39) | <0.001 |
| Sentinel lymph node biopsy | 1.11 (0.80 - 1.55) | 0.536 | 3.80 (2.37 - 6.07) | <0.001 |
| Previous Therapy:  - Neoadjuvant Therapy | 2.00 (1.34- 2.99) | 0.001 | 1.21 (0.71 - 2.06) | 0,475 |
| - Antibody-Based | 2.33 (1.28- 4.26) | 0.006 | 1.37 (0.65 - 2.89) | 0.409 |
| - Radiation | 2.35 (1.43- 3.87) | 0.001 | 2.00 (1.15 - 3.47) | 0.014 |
