# Supplementary material for: Incidence of seroma and postoperative complications after breast surgery before and during the Covid-19 pandemic: results from a retrospective multicenter analysis
Source: BMC Cancer. 2025 Jan 15;25:91. doi: 10.1186/s12885-025-13425-4 (PMC11737024; doi:10.1186/s12885-025-13425-4)
Supplement: Supplementary file 2 — Supplementary Material 2 [file 12885_2025_13425_MOESM2_ESM.docx]

**Supplementary Table A2.:** Bi- and Multivariable analysis using Cox regression model for Time-to-Seroma formation.

| Factors | Bivariate analysis | | Multivariable analysis (n = 921) | |
| --- | --- | --- | --- | --- |
|  | **Crude HR (95%CI)** | **p-value** | **Adjusted HR (95%CI)** | **p-value** |
| SARS- CoV- 2 Pandemic | 1.05 (0.78 - 1.41) | 0.768 | 1.14 (0.82 - 1.57) | 0.440 |
| Age (years): |  |  |  |  |
| <40 | 1 (Ref.) |  | 1 (Ref.) |  |
| 40-49 | 1.88 (1.01 - 3.51) | 0.048 | 1.70 (0.88 - 3.28) | 0.116 |
| 50-59 | 2.36 (1.30 - 4.27) | 0.005 | 2.38 (1.27 - 4.47) | 0.007 |
| 60+ | 2.24 (1.27 - 3.96) | 0.005 | 3.07 (1.66 - 5.69) | <0.001 |
| Body- Mass- Index (BMI): |  |  |  |  |
| <25 | 1 (Ref.) |  |  |  |
| 25<=BMI<30 | 1.30 (0.92 - 1.83) | 0.138 |  |  |
| >=30 | 1.36 (0.93 - 2.00) | 0.115 |  |  |
| Previous Breast Surgery | 1.16 (0.85 - 1.59) | 0.360 |  |  |
| Comorbidities:  - Diabetes mellitus | 1.51 (0.94 - 2.44) | 0.092 |  |  |
| - Smokers | 1.85 (1.24 - 2.75) | 0.002 | 1.43 (0.93 - 2.18) | 0.100 |
| - Autoimmune disease | 1.44 (0.76 - 2.73) | 0.264 |  |  |
| - Allergies | 1.07 (0.67 - 1.72) | 0.776 |  |  |
| Breast Implant Reconstruction | 1.56 (1.10 - 2.21) | 0.013 | 1.53 (1.02 - 2.28) | 0.039 |
| Axillary lymph node dissection | 3.12 (2.21 - 4.41) | <0.001 | 2.96 (1.98 - 4.42) | <0.001 |
| Sentinel lymph node biopsy | 1.09 (0.81 - 1.47) | 0.581 |  |  |
| Previous Therapy:  - Neoadjuvant Therapy | 1.87 (1.32 - 2.64) | <0.001 | 1.21 (0.78 - 1.89) | 0.391 |
| - Antibody-Based | 2.10 (1.27 - 3.47) | 0.004 | 1.19 (0.65 - 2.17) | 0.572 |
| - Radiation | 2.09 (1.37 - 3.17) | 0.001 | 1.61 (1.02 - 2.54) | 0.041 |
